# Study of caudal ropivacaine with or without dexmedetomidine for postoperative analgesia in paediatric genitourinary infraumbilical surgery: a double-blinded randomized controlled trial

**DOI:** 10.1097/MS9.0000000000001919

**Published:** 2024-03-05

**Authors:** Kaushal Tamang, Bidur Kumar Baral, Sadichhya Shah Malla, Brihaspati KC, Sandip Kuikel, Diwan Shrestha, Nibesh Pathak

**Affiliations:** a National Academy of Medical Sciences, Mahaboudhha; b Tribhuvan University Institute of Medicine; c Maharajgunj Medical Campus, Tribhuvan University Institute of Medicine, Kathmandu, Nepal

**Keywords:** caudal block, ropivacaine, dexmedetomidine, infraumbilical surgery, analgesia, postoperative analgesia

## Abstract

Various studies have described the use of Dexmedetomidine with local anaesthetic drugs in caudal blocks for the management of postoperative pain in children. This study was designed to determine the analgesic effect of caudal Dexmedetomidine with Ropivacaine in paediatric genitourinary infraumbilical surgeries. Postoperative analgesic effects of caudal Ropivacaine with or without Dexmedetomidine in paediatric genitourinary infraumbilical were evaluated. This study was a prospective, interventional, comparative study conducted after ethical approval from the institute. Informed expressed consent was taken from each patient’s guardians. The sample size was calculated to be 31 in each group. The two groups were randomly assigned and the intervention involved caudal epidural injection with either Ropivacaine combined with Dexmedetomidine or Ropivacaine with Normal Saline. Children receiving Ropivacaine with Dexmedetomidine had a significantly prolonged duration of analgesia compared to those receiving Ropivacaine alone (840.35 ± 149.97 vs. 412.90 ± 93.46 min, *P* < 0.001). Postoperative rFLACC scores were consistently lower in the Dexmedetomidine group, indicating better pain control (*P* < 0.05 at 6, 12, and 24 h). Total analgesic consumption was lower in the Dexmedetomidine group (500.67 ± 212.92 vs. 741.75 ± 268.06 mg, *P* < 0.01). No significant differences in adverse effects were observed between the groups. The addition of Dexmedetomidine to Ropivacaine in caudal epidural significantly prolongs analgesia, improves pain control, and reduces analgesic consumption in paediatric genitourinary infraumbilical surgeries.

## Introduction

Highlights

- Children receiving Ropivacaine with Dexmedetomidine had a significantly prolonged duration of analgesia compared to those receiving Ropivacaine alone (840.35 ± 149.97 vs. 412.90 ± 93.46 min, *P* < 0.001).
- Postoperative rFLACC scores were consistently lower in the Dexmedetomidine group, indicating better pain control (*P* < 0.05 at 6, 12, and 24 h).
- Total analgesic consumption was lower in the Dexmedetomidine group (500.67 ± 212.92 vs. 741.75 ± 268.06 mg, *P* < 0.01).
- No significant differences in adverse effects were observed between the groups.

Adequate pain control after surgery permits early mobilization, reduces postoperative complications, prevents the development of fear and anxiety, and allows early hospital discharge^1^. Caudal block is one of the modalities of multimodal analgesia. It is a well-established, effective, and relatively safe technique used for intra and postoperative analgesia in children undergoing abdominal and lower-limb surgery.^2^ It provides excellent analgesia, reduces intraoperative anaesthetics drug requirements, ensures pain-free recovery from anaesthesia, decreases stress response, and avoids deleterious adverse effects of narcotic drugs^3^. However, the main disadvantage of the caudal block is its short duration of action with sole local anaesthetics. Even long-acting local anaesthetic drugs such as bupivacaine provide only 4–8 h of analgesia. The insertion of a catheter in caudal space to administer repeated doses or continuous infusions of local anaesthetic drugs is not popular because of concerns of infection^4^. So, various adjuvants like Ketamine^5^, ephedrin^6^, clonidine^7^, opioids^8^ etc. have been used to prolong the duration of analgesia. Morphine and Fentanyl have been used traditionally in combination with a local anaesthetic to achieve prolonged anaesthetic effect. The addition of opioids does provide better analgesia but there is a possibility of an increased incidence of pruritus, urinary retention, nausea, vomiting and respiratory depression^9^. Neostigmine is associated with a higher incidence of vomiting^10^. Similarly, Ketamine and Clonidine are the widely preferred adjuvant to caudal block but have their own number of unpleasant adverse effects. Various studies have described the use of Dexmedetomidine with local anaesthetic drugs in caudal block for the management of postoperative pain in children^11,12^.

Our study aimed to evaluate the postoperative analgesic effects of caudal Ropivacaine with or without Dexmedetomidine in paediatric genitourinary infraumbilical surgery. This involved comparing rFLACC (revised Face, Leg, Activity, Cry, Consolability) score^13,14^ in 24 h of postoperative period, time for first rescue analgesia, total analgesics consumption in 24 h of postoperative period and observed the side effects of study drugs.

## Methods

This was a double-blinded, prospective, randomized clinical trial conducted on 62 children undergoing elective infraumbilical genitourinary surgeries. This work has been reported in line with the CONSORT criteria^15^. Children of either sex aged 2–7 years with American Society of Anesthesiologist (ASA) physical status I or II and undergoing elective genitourinary infraumbilical surgeries were included in the study. The study’s exclusion criteria encompassed patients with injection site infections, bleeding disorders, preexisting psychiatric or neuromuscular diseases, developmental delays or mental retardation, structural deformities of spine or sacrum and allergies to the study drugs.

### Ethical statement

The research was conducted in accordance with Tenets of the Declaration of Helsinki 2013 and approved by the Institutional Review Board, National Academy of Medical Sciences (NAMS) (IRB Approval Number 176/2077/78, IRB approval date: 16 June 2020). Parents or guardians were fully informed about the study’s purpose, duration of participation, nature of procedures, and the option to include or withdraw their child at any point. Written informed consent was obtained from them. Strict confidentiality was upheld, and the patient’s identity was not disclosed. No information, including pictures, videos, or other details, was published without informed written consent. All information was solely used for academic purposes, ensuring no impact on the patient’s physical, mental, or social health.

### Sample size

The sample size calculation was based on the number of rescue analgesia required in the postoperative period. For this study, we took the confidence interval of 95% and power of study 90%. So, Z α =1.96 and Z β =1.28. The effect size (D) and SD^2^ (Pooled SD between the two groups) was taken from a previous study, Anand *et al*.^16^ where the number of rescue analgesia in group receiving Ropivacaine with normal saline mean was ± SD (1.69±0.66) and group receiving Ropivacaine with Dexmedetomidine was ± SD (1.22±0.43).

The number of rescue analgesia in Ropivacaine with normal saline (*D*_1_) =1.69.

The number of rescue analgesia in Ropivacaine with Dexmedetomidine (*D*_2_) =1.22.

The difference between the two groups (*D*) =*D*_2_−*D*_1_ = 0.47.

Standard deviation in group with NS (SD_1_) =0.66.

Standard deviation in group Dexmedetomidine (SD_2_) = 0.43.

Number of patients enroled in the previous study (*n*) = 30.

SD^2^ (Pooled SD between the two groups)

= {(*n*_1_−1) SD_1_^2^ + (*n*_2_−1) SD_2_^2^}/ (*n*_1_+ *n*_2_−2)

= 29(0.66^2^+0.43^2^)/58 =0.30.

The sample size (*n*) = 2 × (*Z* α +*Z* β ) ^2^ × SD^2^/*D* ^2^

= 2 × (1.96+1.28)^2^ × 0.30 /0.47^2^ = 28.51.

Considering 10% drop out, the sample size was calculated to be 31 in each group.

### Interventional details

**Figure 1:**
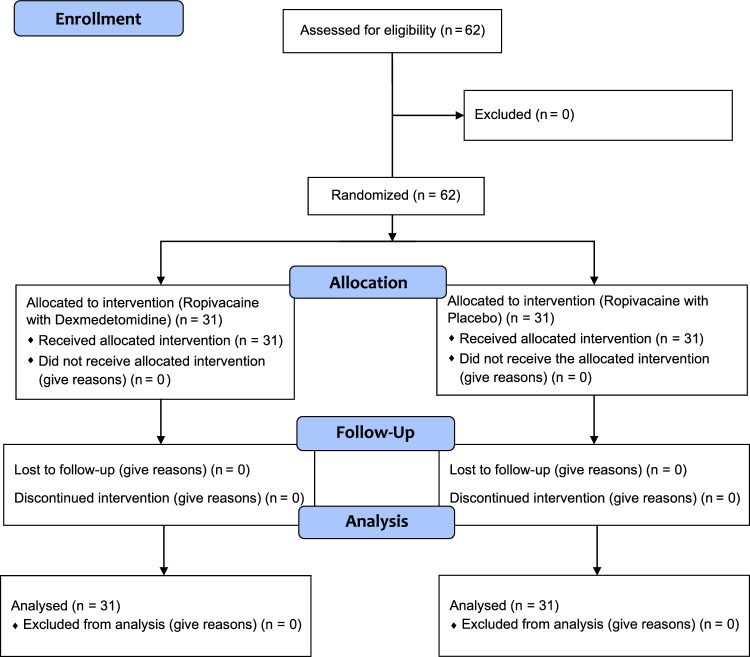
CONSORT flow diagram.

Children characteristics including name, gender, age, body weight and height were recorded. They were randomly assigned to two groups having 31 children in each, by using a computer-generated table of random numbers which was enclosed in a sealed envelope. It was opened by an anesthesiologist who was not involved in the study (Fig. 1).

All children underwent thorough examinations and pre-anaesthetic checkups. Child was kept nil per oral as per ASA guidelines prior to surgery. On the day of surgery in the operating theatre, the child was received, and pre-medication was done with intravenous (IV) Midazolam 0.05 mg/kg. Baseline parameters were monitored and recorded with electrocardiogram (ECG), pulse oximeter (SpO_2_) and noninvasive blood pressure (NIBP). Anaesthesia induction was performed using sevoflurane and oxygen. After reaching the appropriate depth of anaesthesia, a properly sized laryngeal mask airway (LMA) was inserted and maintained using sevoflurane and oxygen, along with assisted ventilation. Following this, the child was positioned laterally, and the skin over the sacrum was disinfected using povidone iodine solution. With aseptic precautions in place, a single-dose caudal epidural injection was administered using a 23-gauge needle. Needle position was confirmed by the “pop” sensation felt upon penetration of the sacrococcygeal ligament, followed by the whoosh test^17^ using 0.5 ml of air. Once blood or cerebrospinal fluid aspiration was confirmed as negative, the caudal medication assigned to each group was administered by an anesthesiologist who was blinded to the drugs. The study drugs were prepared in an unlabelled syringe by an anaesthetist who did not participate in the study and handed over the drug to the anesthesiologist performing the caudal block.

Group A: 1 ml/kg Ropivacaine 0.25% + 1 μg/kg Dexmedetomidine making volume to 0.5 ml.

Group B: 1 ml/kg Ropivacaine 0.25% + along with 0.5 ml of Normal Saline.

The time of the caudal block was noted, and surgery commenced 10 min after the caudal injection. Throughout the procedure, vital parameters including heart rate (HR), ECG, respiratory rate, noninvasive blood pressure (NIBP), and oxygen saturation (SpO2) were continuously monitored. Measurements were recorded before and after induction, after caudal block, after incision, and subsequently every 10 min until the surgery concluded.

At the end of surgery, LMA was removed in deep plane of anaesthesia and patients were shifted to the post-anaesthesia care unit (PACU) for further observation and monitoring. Total duration of surgery was recorded. The primary outcome measured was the duration of analgesia, which was defined as the time elapsed from the administration of the study drugs until the first instance when the rFLACC score (revised Face, Legs, Activity, Cry, and Consolability) reached a greater than or equal to 4^13,14^. FLACC pain scale presented in Table 1 ^14^.

**Table 1:** FLACC Scale

Categories	0	1	2
Face	No particular expression or smile	Occasional grimace or frown, withdrawn disinterested, sad, appears worried	Frequent to constant quivering chin, clenched jaw, distressed looking face, expression of fright/ panic
Legs	Normal position or relaxed, usual tone and motion to limbs	Uneasy, restless, tense, occasional tremors	Kicking or legs drawn up, marked increase in spasticity, constant tremors, jerking
Activity	Lying quietly, normal position, moves easily regular, rhythmic respiration	Squirming, shifting back and forth, tense, tense/ guarded movements, mildly agitated, shallow splinting respirations, intermittent sighs	Arched, rigid or jerking, severe agitation, head banging, shivering, breath holding, gasping, severe splinting
Cry	No cry (awake or sleep)	Moans or whimpers, occasional complaint, occasional verbal outbursts, constant grunting	Crying steadily, screams or sobs, frequents complaints or outbursts, constant grunting
Consolability	Content, relaxed	Reassured by occasional touching, hugging or being talked to, distractible	Difficult to console or comfort, pushing caregiver away, resisting care or comfort measures

Each of the five categories F (face), L (legs), A (Activity), C (Cry), C (Consolability) was scored from 0 to 2 which results in a total score in between zero and ten.

The secondary outcome was side effects of study drugs (vomiting, bradycardia, hypotension and urinary retention).

During the study, adverse events such as nausea, vomiting, bradycardia, respiratory depression, and urinary retention were diligently monitored for a 24-h period, and necessary treatments were provided as required. Postoperative respiratory depression was defined as a respiratory rate below 10 breaths per min or a decrease in oxygen saturation (SpO2) below 95%, necessitating supplementary oxygen. Hypotension was defined as a decrease in blood pressure (BP) by more than 20% from the preoperative values, and bradycardia was defined as a heart rate below 60 beats per min. These conditions were treated with appropriate interventions, such as fluid bolus, Mephentermine or Atropine as deemed necessary.

Postoperative nausea and vomiting were treated with IV Ondansetron 0.1 mg/kg. Postoperative pain was assessed by using the rfLACC pain scale (total score, 0–10). If the rfLACC pain score was greater than or equal to 4 at any time, injection Paracetamol 15 mg/kg was given. If rfLACC pain score still was greater than or equal to 4 after 30 min of injection Paracetamol, then the child was treated with injection Pethidine 0.5 mg/kg. After that, injection Paracetamol at 15 mg/kg was given 6 hourly. Each patient’s pain intensity was assessed at 0, 30 min then 1, 2, 4, 6, 12 and 24 h. The time of the first dose of rescue analgesia given was recorded. The number of doses of rescue medication required in 24 h postoperative was recorded.

### Statistical analysis

The collected data underwent analysis using IBM-SPSS version 21.0. Quantitative variables such as age, weight, surgery duration, pain score, total paracetamol consumption, and time for first rescue analgesia were presented as means ± standard deviation (SD). Qualitative variables like sex, ASA physical status, intraoperative and postoperative complications were presented as numbers or frequency (%). For quantitative variables with a normal distribution, Student’s unpaired *t*-test was used; otherwise, the Mann–Whitney test was applied for abnormal distributions. Qualitative variable comparisons utilized Pearsons’s χ^2^ test or Fisher’s exact test as appropriate. A *p* value less than 0.05 was considered statistically significant.

## Results

A total of 62 children (31 in each group) scheduled for elective genitourinary infraumbilical surgeries were enroled in the study. Both groups were comparable regarding age, gender, weight, type of surgery and duration of surgery. (Table 2) The types of surgery in both the groups were also comparable. They were not statically significant (Table 3).

**Table 2:** Demographic profiles of children

Patient characteristics	Group A (*n*=31) mean ± SD	Group B (*n*=31) mean ± SD	*P*
Age (years)	4.92 ± 2.54	4.54 ± 2.42	0.552^a^
Sex (M/F)	26/5	23/8	0.623^b^
Weight (kg)	15.93 ± 5.73	15.27 ± 5.59	0.647^a^
Duration of surgery (min)	54.22 ± 22.3	60.45 ± 28.62	0.343^a^

F, female; M, male.

a Students *t*-test.

b χ^2^ test.

**Table 3:** Comparisons of type of surgery

Type of surgery	Group A (*n*=31)	Group B (*n*=31)	*P*
Herniotomy	10	7	0.40
Hydrocele	6	5	0.74
Circumcision	6	8	0.55
Urethroplasty	4	5	0.45
Orchidectomy	2	3	0.56
Orchidopexy	3	3	1.00

### Duration of analgesia

The duration of analgesia was defined as time between administration of caudal block until the rFLACC score reached greater than or equal to 4. The mean duration of analgesia was 840.35 ± 149.97 min in group A and 412.90 ± 93.46 min in group B and *p* value was 0.0002. This shows the duration of analgesia was significantly prolonged by the addition of Dexmedetomidine to Ropivacaine.

### Postoperative pain assessment (rFLACC score)

Postoperative pain was assessed by using the rfLACC scale (total score, 0–10). During the first 4 h after operation, all the patients in both the groups had adequate analgesia (rFLACC score < 4). After that, patients in Group B receiving Ropivacaine only, achieved significantly higher rFLACC pain score compared to the patients in Group A receiving Ropivacaine with Dexmedetomidine and the difference was statistically significant (Table 4).

**Table 4:** Postoperative pain assessment (rFLACC score)

Time	Group A (*n*=31) mean ± SD	Group B (*n*=31) mean ± SD	*P*
30 min	1± 1.09	1.09 ± 1.27	0.749
1 h	1.19± 0.98	1.35 ± 1.37	0.597
2 h	1.93±1.26	1.93 ± 1.45	1.00
4 h	0.81±1.32	1.51 ± 1.50	0.053
6 h	3±1.15	4 ± 1.75	0.010^a^
12 h	5.19±1.32	4.12 ± 0.84	0.0004^a^
24 h	4.09±0.47	5.09 ± 1.32	0.0002^a^

a *P* value < 0.05, Students *t*-test.

### rFLACC score and subgroup analysis at different postoperative period:

**Figure 2:**
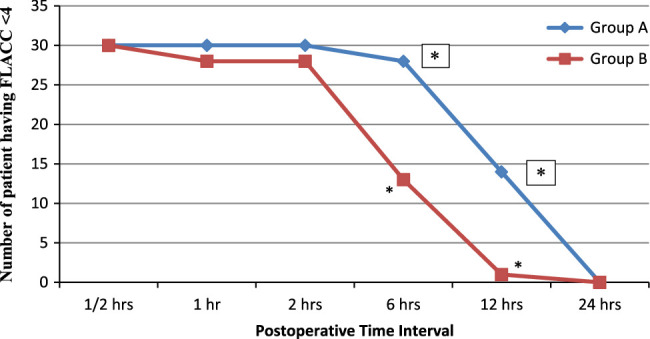
Assessments of rFLACC Pain Score less than 4 at various time intervals. *p value less than 0.05 at 6 and 12 postoperative hours.

In the present study, we evaluated the postoperative pain by rFLACC Score and also subgroup analysis was done by dividing the study population into two groups having rFLACC less than 4 or greater than or equal to 4 at different time intervals of first 24 h of postoperative periods. In the first 4 h of postoperative period the rFLACC score was found less than 4 in both groups, so none of the children required rescue analgesia. Then the rFLACC score was found to be increased at different time intervals of postoperative periods. At 6 h of postoperative period, 28 (90.32%) children had rFLACC score less than 4 in group A whereas 13 (41.9%) of the patients in group B had rFLACC score less than 4 with *p* value of 0.0001. Similarly at 12 h, 14 (45.1%) children in group A and only 1 (3.2%) of children in group B had rFLACC score less than 4 with *p* value of 0.042. However at 24 postoperative hours, none of the patients had rFLACC score less than 4 in both the groups (Fig. 2).

### Analgesics consumption in first 24 h postoperative period

The total Paracetamol requirement in Group B patients was 741.75 ± 268.06 mg whereas in Group A was 500.67 ± 212.92 mg in first 24 h of postoperative (*P* value is <0.01). This shows that the Paracetamol consumption was significantly lower in the group of children receiving Dexmedetomidine with Ropivacaine. The maximum number of doses of rescue analgesia requirements was found to be three doses during the study periods. In children belonging to group A, none of the patients required 3 doses of rescue analgesia, 2 (6.4%) patients required 2 doses, 23 (74.1%) patients required only one dose and 6 (19.5%) patients did not require any rescue analgesia during the first 24 hours of postoperative period. Whereas in group B, 14 children (45.16%) required 3 doses of rescue analgesia, 12 (38.70%), required 2 doses, 3 (9.67%) children needed 1 dose and 2 (6.47%) children did not require any rescue analgesia during the first 24 h of postoperative period. This result shows the total number of doses of rescue analgesia required was less in children receiving Dexmedetomidine with Ropivacaine as compared to children receiving Ropivacaine only.

### Side effects of study drugs

In group A, 3 (9.2%) children and in group B, 4 (12.9%) children vomited. The incidence of vomiting was not statistically significant between the two groups. Hypotension was found in 2 (6.6%) children in both groups and none of the children in both the groups had clinically significant bradycardia, respiratory depression, and urinary retention.

## Discussion

Dexmedetomidine is pharmacologically active d-isomer of Medetomidine. It is claimed to possess anxiolytic, sedative, sympatholytic and analgesic properties without respiratory depression and undesirable side effects^4^. It can be used to enhance the effects of local anaesthetics without increasing incidence of the side effects^18^. Its effect is extrapolated from studies of clonidine and it is an effective sedative and analgesic agent than Clonidine without undesirable cardiovascular effects, due to lack of α1 receptor activation even with higher doses^19^. Dexmedetomidine is commonly employed in paediatrics anaesthesia as pre-medication, procedural sedation, ICU sedation, and adjuvants agents in central and peripheral nerve block. Its use is currently off-label in spite of its strong evidence in paediatric anaesthesia^20,21^.

It is assumed that the enhancement of caudal block with addition of Dexmedetomidine are primarily attributable to its direct peripheral action on nerves in block, rather than dexmedetomidine’s central activity following absorption through the block site into systemic circulation, which results in systemic effects^22^.

The precise mechanism by which intrathecal α2 adrenoreceptor agonists prolong the motor and sensory block of local anaesthetics is not yet fully understood. It may be attributed to an additive or synergistic effect resulting from the distinct mechanisms of action^23^. However many recent research have observed a significant synergistic effect, resulting in a more profound and extended blockade with improved hemodynamic stability compared to using these drugs individually^24,25^. The extensive distribution of α-2 adrenergic receptors in both the central and peripheral nervous systems is pivotal in mediating the effects of neuraxial dexmedetomidine. Dexmedetomidine causes local vasoconstriction and hyperpolarization, delaying the absorption of local anaesthetics like ropivacaine and prolonging their effects^26^. Analgesia results from direct stimulation of pre-synaptic and post-synaptic α2 adrenoreceptors in the dorsal grey matter of spinal cord which inhibits the release of nociceptive neurotransmitters. It is correlated with the concentration of dexmedetomidine in the cerebrospinal fluid but not that in the plasma^4,27^. Intrathecal dexmedetomidine inhibits spinal ERK1/2 signalling which results in potent analgesia effects in a manner dependent on α2 receptors^28^. Thus further research should be done about the pharmacodynamics of this mixture before using them clinically.

Abdallah and Brull’s 2013 meta-analysis suggested that dexmedetomidine could enhance the efficacy of local anaesthetics in intrathecal and peripheral nerve blockade, but safety remained inconclusive^29^. In a subsequent reevaluation in 2017, they found that perineural dexmedetomidine was considered safe, providing updated and potentially more reassuring insights into its safety profile^30^. Hou and colleagues suggested that intrathecal dexmedetomidine at low doses (0.75:1.50 µg/kg) can alleviate pain without causing neurotoxicity^31^. Konakci and colleagues study in rabbits suggested that an epidural injection of dexmedetomidine at a dose of 4.8–6.1 μg kg^−1^ and a concentration of 10 μg ml^−1^ could potentially cause neurotoxicity and demyelination of oligodendrocytes in white matter. The study concluded that while dexmedetomidine doesn’t have apparent motor and sensory effects, it may pose a risk to the myelin sheath when administered epidurally^27^. However several studies in children have found that administering dexmedetomidine through neuraxial routes at doses not exceeding 2 μg kg^−1^ and concentrations of no more than 2 μg ml^−1^ does not lead to neurotoxicity^11,32^. We opted for a dexmedetomidine volume of 1 μg kg^−1^, aligning with the dosage used in comparable studies, given the established safety regarding neurotoxicity in children.

In our study, duration of postoperative analgesia was significantly prolonged in children receiving Ropivacaine with Dexmedetomidine. These results are concurrent to findings in a study conducted by Jain *et al*.^33^ found that duration of analgesia was 797.00 ± 59.20 minutes in group receiving Ropivacaine with Dexmedetomidine whereas 3.63.30 ± 31.44 minutes in group receiving Ropivacaine only. Another study done by Kamal *et al*.^34^ reported that the mean duration of analgesia was 390 (414.95–483.05) min in Ropivacaine group as compared with 750 (771.08–926.92) min in group receiving Ropivacaine with Dexmedetomidine. Anand *et al*.^16^ found that the duration of analgesia was of 5.5 h (4.97–6.03) in Ropivacaine group compared with 14.5 h (13.90–15.09) in group receiving Ropivacaine with Dexmedetomidine. Similar results were found in a study done by Gupta *et al*.^35^ where the mean duration of analgesia was 780.29 ± 71.21 min in patient receiving Ropivacaine with Dexmedetomidine compared to 654.20 ± 78.38 min in group receiving Ropivacaine and Tramadol.

Our findings of decreased severity of pain in Ropivacaine with Dexmedetomidine are comparable with the findings of similar studies. In our study, children of both the groups had adequate analgesia (rFLACC score < 4) during the first 4 h after operation, then the number of patients with adequate analgesia declined in group B and achieved significantly higher rFLACC score as compared to group A. The number of children having pain (rFLACC score ≥4) increased rapidly in Group B at 6, 12 and 24 h postoperatively in comparison with group A. At 6 h of postoperative period, 28 (90.32%) children had rFLACC score less than 4 in group A whereas 13 (41.9%) of the patients in group B had rFLACC score less than 4. Similarly at 12 h, 14 (45.1%) children in group A and only 1 (3.2%) of children in group B had rFLACC score less than 4. At 24 postoperative hours none of the patients had rFLACC score less than 4 in both the groups.

Kamal and colleagues found both groups had adequate analgesia for first 4 h, then at 6 h, 60% of patients in Ropivacaine group achieved a FLACC score greater than or equal to 4 whereas none of the patients achieved FLACC score greater than or equal to 4 in group with Ropivacaine with Dexmedetomidine group. After 18 h of postoperative period, 60% of patients in group receiving Ropivacaine with Dexmedetomidine had achieved FLACC score of greater than or equal to 4^34^. Anand and colleagues found that patients with only Ropivacaine achieved significantly higher FLACC score compared with patients receiving Ropivacaine with Dexmedetomidine. Twenty out of 30 children achieved a FLACC score of more than four at six hours of postoperative period. Compared with Ropivacaine with Dexmedetomidine, there were no patients achieved rFLACC greater than or equal to 4 at 16 h of postoperative period^16^. In another study conducted by Bharti *et al*.^36^ all patients in the plain Ropivacaine group had FLACC pain score greater than 4 in first 6 h of postoperative period, while none of the patient receiving Ropivacaine with Dexmedetomidine achieved FLACC greater than 4.

In our study, the total requirement of paracetamol consumed was more in Group B with the mean of 741.75 ± 268.06 mg than in Group A patients with a mean of 500.67 ± 212.92 mg and the difference was statistically significant. Singh *et al*.^37^ discovered that the mean total analgesic intake with Levobupivacaine alone was 392.3187.5 mg, whereas the group with Levobupivacaine plus Dexmedetomidine used 268.9111.8 mg, with the difference between the two groups being statistically significant. Dimitri and colleagues conducted an extensive study on ibuprofen use in the paediatric population, revealing its superior efficacy to acetaminophen. and comparable efficacy to the acetaminophen-codeine combination in managing acute musculoskeletal pain, showcasing a favourable safety profile. Importantly, the findings suggested a reconsideration of ibuprofen’s role in managing postoperative pain^38^.

During the study period postoperative nausea and vomiting (PONV) and hypotension were observed, where 3 (9%) patients in group A and 1 (2.9%) patient in group B vomited. None of the patients in either of the groups had clinically significant respiratory depression, bradycardia, and urinary retention. Similar results were shown by previously published articles^33,34^.

There are few limitations of our study. Assessment of duration and power of the motor block, postoperative sedation and emergence time were not evaluated in this study. Additionally, study could not assess the severity of surgical trauma which may lead to variability in pain severity and postoperative pain score beyond 24 h. Finally, although we did not experience a single block failure, performing the procedure under ultrasound guidance would have been safer and more precise.

## Conclusion

Dexmedetomidine (1 μg/kg) with 0.25% Ropivacaine in single shot caudal block prolongs the duration of analgesia, provides effective analgesia and reduces requirement for rescue analgesic consumption in the first 24 h of postoperative period. Thus, Dexmedetomidine, which enhances the effects of local anaesthetics can be used as an adjunct for analgesic agent in paediatric genitourinary infraumbilical surgeries.

## Ethics statement

The Institutional Review Board and Ethical Committee of National Academy of Medical Science (NAMS) approved the conduction of this study (Approval number: 179/2077/78). The protocol was registered in Clinicaltrials.gov and it is public. (ClinicalTrials.gov ID: NCT05979558).

## Consent

Written informed consent was obtained from the patient’s parents/legal guardian for publication and any accompanying images. A copy of the written consent is available for review by the Editor-in-Chief of this journal on request.

## Source of funding

Not applicable.

## Author contribution

K.T., B.K.B., S.S.M.: conceptualization; data curation; investigation; methodology; project administration; resources; validation; writing—review and editing. B.K.C.: Conceptualization; Data curation; Investigation; Methodology; Project administration; Resources; validation; writing—review and editing; formal analysis; Writing—original draft. S.K., D.S., N.P.: formal analysis; methodology; validation; writing—original draft; writing—review and editing. All authors have read and approved the final version of the manuscript. K.T. had full access to all the data in this study and takes complete responsibility for the integrity of the data and the accuracy of the data analysis.

## Conflicts of interest disclosure

The authors declare no conflicts of interest.

## Research registration unique identifying number (UIN)

The protocol was registered in Clinicaltrials.gov and it is public. (ClinicalTrials.gov ID: NCT05979558).

## Guarantor

Dr Kaushal Tamang.

## Data availability

All the data can be obtained upon reasonable request to the corresponding author.

## Provinence and peer review

Not Applicable.

## Transparency statement

The lead author affirms that this manuscript is an honest, accurate, and transparent account of the study being reported; that no important aspects of the study have been omitted; and that any discrepancies from the study as planned (and, if relevant, registered) have been explained.
